# Myoepithelioma of the Nasal Septum: A Rare Case of Extrasalivary Gland Involvement

**DOI:** 10.1155/2017/7057989

**Published:** 2017-01-10

**Authors:** Gustavo Barreto da Cunha, Tatiane Costa Camurugy, Thiago Cavalcante Ribeiro, Nara Nunes Barbosa Costa, Amanda Canário Andrade Azevedo, Eriko Soares de Azevedo Vinhaes, Nilvano Alves de Andrade

**Affiliations:** Santa Casa de Misericórdia da Bahia, Hospital Santa Izabel, Salvador, BA, Brazil

## Abstract

*Introduction*. The myoepithelioma is a rare benign tumor, most frequently found in the salivary glands. The extrasalivary gland involvement is even rarer and few cases involving the nasal cavity have been reported in the literature.* Case Report*. MES, a 54-year-old woman, complaining of progressive nasal obstruction and mild epistaxis through the right nostril which had developed 1 year previously. Computed tomography scan showed tumor with heterogeneous contrast enhancement occupying the right nasal cavity, moving contralaterally in the nasal septum. Excisional biopsy was performed through endoscopic surgery of the mass that was inserted at the nasal septum. Pathological and immunohistochemical exams concluded myoepithelioma.* Discussion*. The main symptoms of nasal myoepitheliomas are nasal obstruction and epistaxis. Immunohistochemistry is necessary to confirm the diagnosis, typically positive for cytokeratin and S-100, calponin, smooth muscle actin, myosin, vimentin, glial fibrillary acidic protein (GFAP), and carcinoembryonic antigen. The main marker for myoepithelioma is the S-100 protein. In our case, it was positive for cytokeratin, S-100, calponin, actin smooth muscle, and GFAP. In all cases reported in the literature surgical treatment was performed and the recurrence was associated with incomplete tumor resection.* Final Comments*. The myoepithelioma is a rare differential diagnosis of nasal tumors and its treatment is the total lesion excision.

## 1. Introduction

Myoepithelioma is a rare benign tumor, most frequently found in the salivary glands, representing, however, only 1% of all tumors of these glands. In the major salivary glands there is a predilection for the parotid and in the minor salivary glands for the palate and oral cavity [1, 2]. The extrasalivary involvement is even rarer and just few cases involving the nasal cavity have been reported in the literature. This is a case of myoepithelioma of the nasal septum and the discussion about its diagnosis and treatment.

## 2. Case Report

**Figure 1:**
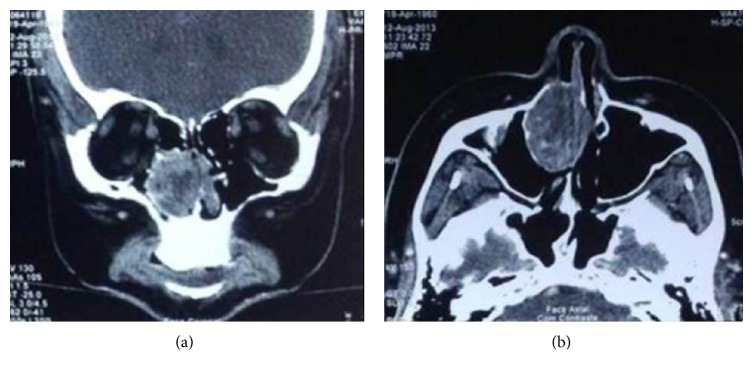
Computed tomography. Coronal (a) and axial (b) planes demonstrate heterogeneous contrast enhancement by the tumor.

**Figure 2:**
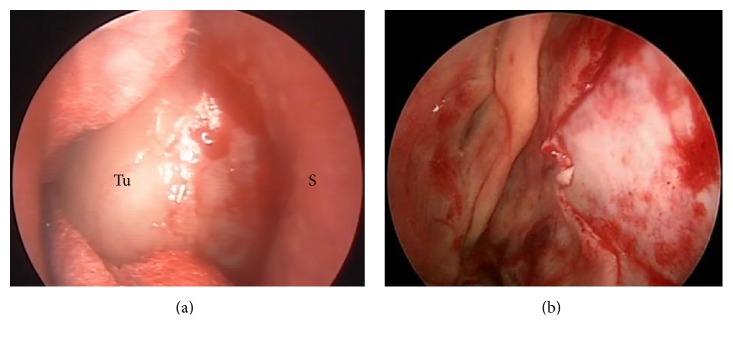
Endoscopic appearance. Preoperative (a) and immediately after resection (b). Tu = tumor. S = nasal septum.

MES, a 54-year-old woman, presented at our hospital complaining of progressive nasal obstruction and mild epistaxis through the right nostril which had developed 1 year previously. She denied smoking and had no known toxic exposure or family history of neoplasms. Nasofibroscopy revealed irregular vascular lesion which occupied the right nasal cavity almost completely. Computed tomography (CT) scan showed heterogeneous contrast enhancement by the tumor, which displaced contralaterally the nasal septum (Figure 1). Complete endoscopic resection of the lesion that had originated from the nasal septum was performed. Ipsilateral septal mucosa and septal cartilage as surgical margin were removed, opting for the preservation of the contralateral mucosa, which was found apparently free of disease (Figure 2).

The material that consisted of several brown tissue fragments measuring together 6,0 × 3,0 × 1,0 cm was sent for pathological examination, which showed morphological features compatible with myoepithelioma, despite the focal presence of chondroid matrix and occasional ductal structures. Immunohistochemistry presented positivity for glial fibrillary acidic protein (GFAP), smooth muscle actin, cytokeratin (AE1AE3 and MNF116), calponin, P63, S100, and CD99. The septal margin was found to be affected by the tumor but was chosen for follow-up, and after two years of follow-up there was no evidence of tumor recurrence or metastasis.

## 3. Discussion

Nasal cavity and paranasal sinus tumors are very infrequent, accounting for less than 1% of all head and neck tumors [3]. Nasal mucosa contains mucous glands, minor salivary glands, melanocytes, and olfactory neuroepithelial cells [4]. Myoepithelioma is a benign slow-growing tumor of the salivary glands described by Sheldom in 1943 [5]. The main symptoms of nasal myoepitheliomas are nasal obstruction, due to tumor growth, and epistaxis [6–8], symptoms presented by the patient reported in this paper. The CT does not have a well-defined standard, but it is an important test for therapeutic planning. No relationship was found between sex, age, and injury presented.

**Figure 3:**
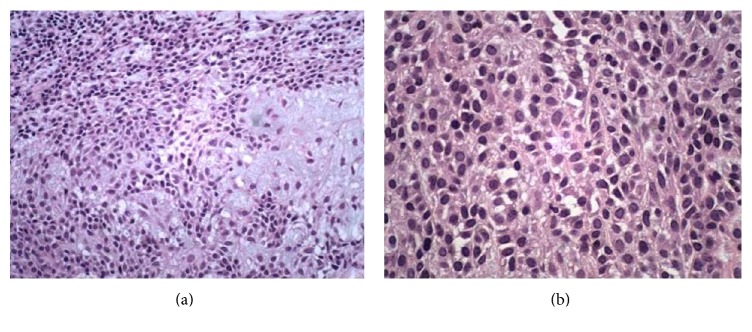
Microscopic view, H and E stain, 200x (a) and ×400 (b). Typical plasmacytoid myoepithelial cells among myxoid stroma.

Myoepithelioma is composed of myoepithelial cells and may have pattern of solid growth, myxoid or reticular. The cell types found are fusiform, plasmacytoid, epithelioid, and clear cells. The pattern of growth and cell type does not change the prognosis of the disease [9–12]. This is a tumor classically defined as not having a ductal differentiation, but several authors have adopted a less rigid definition, including the presence of a small number of ducts (presence in less than 5% of the examined field) as well as in the present case (Figure 3) and also in other reported cases of myoepitheliomas of the nasal cavity [6–8, 13]. The main differential diagnosis is the pleomorphic adenoma, but it presents myoepithelial cells in varied proportions and ductal formations are numerous [9, 10, 12].

Immunohistochemistry is necessary to confirm the diagnosis, typically positive for cytokeratin, S-100, calponin, smooth muscle actin, myosin, vimentin, glial fibrillary acidic protein (GFAP), and carcinoembryonic antigen [14, 15]. The main marker for myoepithelioma is the S-100 protein and the diagnosis will hardly be made if negative [12]. The leiomyomas and squamous cell cancers are negative for S-100 protein. The schwannomas are positive but have their own histological features [1]. In our case, it was positive for cytokeratin, S-100, calponin, smooth muscle actin, and GFAP.

In all cases reported the surgical treatment was performed and the recurrence has been associated with incomplete resection of tumor, which proves the aggressive biological behavior of this tumor [16, 17]. Histologically this tumor is well circumscribed, so complete excision is possible [18]. This neoplasm is not radio- or chemosensitive and it needs a long-term follow-up since recurrence varied from 35% to 50% and metastasis rates from 8.1% to 25% in different reports [19]. This case had neither recurrence nor metastasis two years after surgery.

There are very few cases of myoepithelioma involving the nasal cavity reported in the literature. However in the last few years the publications of its extrasalivary gland involvement increased substantially, which makes the authors question if it is an underdiagnosed disease. The advances in pathology and immunohistochemistry knowledge and techniques may help us to diagnose it more and more commonly in the future. Therefore, although it is rare, we have to consider it as a differential diagnosis of nasal tumors.
